# New-Onset Occipital Lobe Epilepsy in an Elderly Patient With Visual Hallucinations and Hemianopia

**DOI:** 10.7759/cureus.64903

**Published:** 2024-07-19

**Authors:** Katarina Milosavljevic, Yong Eun, Pooja Roy, Salama Fawzy

**Affiliations:** 1 Anatomy, Touro College of Osteopathic Medicine, New York City, USA; 2 Internal Medicine, Harlem Hospital Center, New York City, USA; 3 Neurology, Harlem Hospital Center, New York City, USA

**Keywords:** electroencephalography, migraines with visual aura, hemianopia, visual hallucination, occipital lobe epilepsy

## Abstract

Occipital lobe epilepsies (OLEs) are a subset of epileptic disorders manifesting predominantly with visual and oculomotor abnormalities that are often misdiagnosed due to similarities with migraines with visual aura and other central nervous system (CNS) pathologies. This case study describes an 88-year-old male with a three-week history of intermittent kaleidoscopic visual phenomena, accompanied by blurring of vision and altered level of consciousness. Neurological examination revealed right homonymous hemianopsia and focal neurological deficits, including forced right gaze preference and nystagmus. Diagnostic modalities, MRI and MRA, ruled out ischemic stroke but indicated mild to moderate cerebral atrophy and chronic microvascular ischemic changes. The patient exhibited a seizure episode characterized by right-sided gaze preference and altered consciousness. Postictally, transient right homonymous hemianopsia was observed, consistent with Todd's phenomenon. Treatment with intravenous levetiracetam and lorazepam led to a reduction in seizure frequency. This case highlights the importance of comprehensive evaluation in distinguishing OLEs from other conditions with similar visual presentations like migraine with aura or occipital lobe stroke being more predominant.

## Introduction

Occipital lobe epilepsies (OLEs) represent a relatively uncommon subgroup of epileptic disorders, accounting for an estimated 5% to 10% of epilepsies. These conditions are characterized by distinctive visual manifestations that can often be confused with other central nervous system (CNS) pathologies, such as migraines with visual aura, or even ophthalmological disorders [1]. This potential for misdiagnosis underscores the necessity for a thorough diagnostic approach to ensure timely and appropriate patient care. Patients with OLEs typically do not have a medical history of migraines, either with or without aura [2,3]. The visual hallucinations associated with OLEs frequently involve vivid, multicolored, circular, or spherical patterns that evolve dynamically [4]. These complex visual phenomena are more indicative of CNS involvement rather than ophthalmologic conditions. Another distinguishing feature of OLEs is the possible altered level of consciousness during episodes, a contrast to the preserved awareness observed in migraine sufferers. The duration of visual symptoms in OLEs typically spans from a few seconds up to three minutes [2]. These seizures often manifest in multiple daily or weekly clusters and may include preceding or subsequent blurring of vision [2-4]. In contrast, the visual auras of migraines tend to persist for 15 to 30 minutes and generally have a static quality. With a high index of clinical suspicion, the diagnosis of OLEs can be corroborated by diagnostic modalities such as electroencephalography (EEG), and neuroimaging with computed tomography (CT) or magnetic resonance imaging (MRI) [5].

## Case presentation

An 88-year-old male with a medical background significant for hypertension, hyperlipidemia, and diabetes mellitus (not on any medications) presented with a three-week history of unusual visual disturbances. His home medications were Amlodipine 10 mg once daily and Atorvastatin 40 mg nightly. The patient described intermittent, kaleidoscopic visual phenomena, predominantly featuring red, blue, and yellow hues, manifesting as mushroom shapes and twirling floors that evolved into flowers with various petals. These symptoms were noted on the right side of his visual field, each lasting one to three minutes, and were often accompanied by a blurring of vision persisting for approximately 15 minutes before normalization. Neurological assessment revealed no focal deficits and a normal gait. His laboratory findings on presentation were within normal limits (Table 1). Ophthalmological evaluation, while the patient was experiencing visual deficit, identified a right homonymous hemianopsia; extraocular movements were intact (Table 2).

**Table 1:** Basic laboratory finding on presentation

Component ref. range and units	
WBC (white blood cell) 4.80 - 10.80 x10(3)/mcL	11.55
RBC (red blood cell) 4.70 - 6.10 x10(6)/mcL	3.05
HGB (hemoglobin) 14.0 - 18.0 g/dL	9.3
HCT (hematocrit) 42.0-52.0%	27.1
Sodium 136-145 mmol/L	139
Potassium 3.5-5.1 mmol/L	4.3
Chloride 98-107 mmol/L	100
CO2 (bicarbonate) 22-29 mmol/L	27
BUN (blood urea nitrogen) 7-18 mg/dL	14
Creatinine 0.7-1.2 mg/dL	1.2

**Table 2:** Detailed ophthalmologic examination done on admission showing finding consistent with right homonymous hemianopsia PERLA = pupils are equal, round, and reactive to light and accommodation, APD = Afferent Pupillary Defect, J1 = Patients can read 4pt font size CC = choroidal conus/with correction

Ophthalmology exam finding	Right	Left
Pupils	PERLA, no APD	PERLA, no APD
Visual Acuity (Snellen-Linear)		
Near CC (with correction)	J1	J1
Tonometry Pressure (Tonopen Avia)	19	20
Visual Field restriction	Total superior nasal, inferior nasal deficiencies	Total superior nasal, inferior nasal deficiencies
Slit Lamp Examination		
Lids/Lashes	Normal	Normal
Conjunctiva/Sclera	White and quiet	White and quiet
Cornea	Clear	Clear
Anterior Chamber	Deep and quiet	Deep and quiet
Iris	Round and reactive	Round and reactive
Lens	Clear	Clear
Vitreous	Normal	Normal

**Figure 1:**
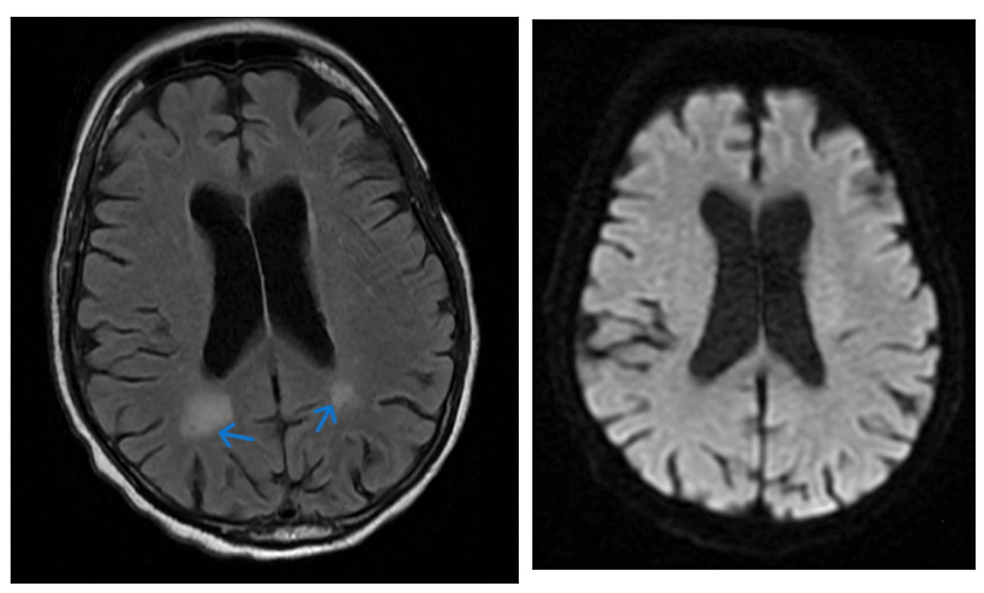
MRI T2 FLAIR images (blue arrow left) demonstrate areas of hyperintensity in the periatrial white matter bilaterally. There is no signal abnormality within the corpus callosum or along the callosal septal interface. DWI image (right) demonstrates no evidence of enhancing parenchymal lesion or abnormal leptomeningeal enhancement and shows mild to moderate cerebral atrophy and chronic microvascular ischemic changes. MRI: Magnetic resonance imaging, T2 FLAIR: T2 and fluid-attenuated inversion recovery

The patient was unaware of his visual deficits, and a point-of-care ocular ultrasound ruled out retinal detachment or vitreous hemorrhage. During our examination in the emergency department, the patient experienced a total of six episodes characterized by a forced right gaze preference with concomitant right-beating nystagmus on the left lateral gaze, which resolved on upward gaze. The episodes, lasting one to two minutes, were also accompanied by an altered level of consciousness with sluggish responsiveness. Post-episode, the patient reported transient right homonymous hemianopia. These episodes were repeated multiple times during his stay. Initial computed tomography (CT) head showed no evidence of acute intracranial hemorrhage or fracture, mild of moderate cerebral volume loss with no evidence of mass, hydrocephalus or cortical infarct. Electroencephalogram (EEG) showed no epileptiform activity. An MRI indicated hyperintensity in the periatrial white matter area, mild to moderate cerebral atrophy and chronic microvascular ischemic changes (Figure 1).

MRA showed no significant large vessel occlusions and carotid duplex ultrasonography found no notable stenosis. Given the recurrent episodes, the patient was loaded with levetiracetam 2000 mg and maintained with intravenous levetiracetam 750 mg twice daily. Lorazepam 2 g as needed was used for seizure cessation. Following a progressive improvement over four days, with a noted reduction in the frequency of episodes, the patient was discharged with Levetiracetam 1000 mg twice daily and scheduled for outpatient neurological follow-up in four weeks.

To assist in the identification and management of similar cases, it is important to note the sequence of symptom presentation and diagnostic findings. Initially, the patient experienced visual disturbances characterized by kaleidoscopic visuals predominantly in red, blue, and yellow, appearing as mushroom shapes and evolving floral patterns. These episodes were brief, lasting one to three minutes, but the visual blurring persisted for about 15 minutes. A timeline of symptoms alongside corresponding diagnostic findings, such as the right homonymous hemianopsia detected during the ophthalmological evaluation, can aid in distinguishing OLE from similar presentations. Understanding the typical progression and response to treatment can also guide management in new cases. Therefore, we recommend a systematic approach to documenting symptom onset, progression, and response to interventions in clinical practice.

## Discussion

In this case, our differential diagnoses included left occipital lobe ischemia, focal seizure with altered level of consciousness, and migraine with visual aura. The final diagnosis was determined to be an OLE with an altered level of consciousness accompanied by postictal visual impairment. The absence of a migraine history, coupled with the unlikely presentation of homonymous hemianopsia as a migraine symptom, decreased the probability of migraine with visual aura. Typically, patients with migraines can recognize visual deficits, which was not the case here [3]. The patient's communication difficulties, where he could only articulate in brief phrases with notable pauses, suggested an altered level of consciousness consistent with seizure activity. The MRI did not reveal any signs of ischemia; thus, the persistent right homonymous hemianopsia was attributed to a postictal state - commonly known as Todd’s phenomenon - rather than ischemic events [6]. This phenomenon can be explained by the temporary stunning of neurons, which fail to maintain ion gradients necessary for light detection after intense electrical activity and ATP depletion. The diagnosis hinged on the observed seizure episode: the patient was initially speaking fluently but then exhibited a marked reduction in speech fluency and developed a right-sided gaze preference. Additionally, the fluctuating nature of the patient's right homonymous hemianopsia, which improved during seizure-free intervals and worsened postictally, further supported the seizure diagnosis.

## Conclusions

This case represents a unique instance of occipital seizures that can be easily mistaken for visual migraines without headache, also known as acephalgic migraine visual aura. However, the distinguishing feature in this patient was the extended duration of the visual deficits, which is atypical for migraines. The ophthalmologic examination findings, combined with the patient's presentation of Todd's phenomena, contributed to the clear diagnosis for our case. 
